# 4-Phenylbutyric Acid (4-PBA) Derivatives Prevent SOD1 Amyloid Aggregation In Vitro with No Effect on Disease Progression in SOD1-ALS Mice

**DOI:** 10.3390/ijms23169403

**Published:** 2022-08-20

**Authors:** Leenor Alfahel, Shirel Argueti-Ostrovsky, Shir Barel, Mahmood Ali Saleh, Joy Kahn, Salome Azoulay-Ginsburg, Ayelet Rothstein, Simon Ebbinghaus, Arie Gruzman, Adrian Israelson

**Affiliations:** 1 Department of Physiology and Cell Biology and The Zlotowski Center for Neuroscience, Ben-Gurion University of the Negev, P.O. Box 653, Beer Sheva 8410501, Israel; 2 Department of Chemistry, Faculty of Exact Sciences, Campus Ramat-Gan, Bar-Ilan University, Ramat-Gan 5290002, Israel; 3 Institute of Physical and Theoretical Chemistry, TU Braunschweig, Rebenring 56, 38106 Braunschweig, Germany

**Keywords:** amyotrophic lateral sclerosis (ALS), mutant SOD1, misfolded proteins, aggregation, chemical chaperones

## Abstract

Amyotrophic lateral sclerosis (ALS) is a fatal neurodegenerative disease characterized by the degeneration of motor neurons. Mutations in the superoxide dismutase (SOD1) gene, causing protein misfolding and aggregation, were suggested as the pathogenic mechanisms involved in familial ALS cases. In the present study, we investigated the potential therapeutic effect of C4 and C5, two derivatives of the chemical chaperone 4-phenylbutyric acid (4-PBA). By combining in vivo and in vitro techniques, we show that, although C4 and C5 successfully inhibited amyloid aggregation of recombinant mutant SOD1 in a dose-dependent manner, they failed to suppress the accumulation of misfolded SOD1. Moreover, C4 or C5 daily injections to SOD1^G93A^ mice following onset had no effect on either the accumulation of misfolded SOD1 or the neuroinflammatory response in the spinal cord and, consequently, failed to extend the survival of SOD1^G93A^ mice or to improve their motor symptoms. Finally, pharmacokinetic (PK) studies demonstrated that high concentrations of C4 and C5 reached the brain and spinal cord but only for a short period of time. Thus, our findings suggest that use of such chemical chaperones for ALS drug development may need to be optimized for more effective results.

## 1. Introduction

Amyotrophic lateral sclerosis (ALS) is a rapidly progressive and fatal neurodegenerative disease characterized by the degeneration of the upper and lower motor neurons (MNs) in the brain and spinal cord [1]. About 10% of ALS cases are familial, most of which are inherited in a dominant manner [2], and approximately 20% of these familial cases are attributed to mutations in the superoxide dismutase 1 (SOD1) gene [3]. To date, more than 180 different SOD1 variants have been associated with ALS [4].

Although the exact mechanisms underlying MN degeneration remain to be elucidated [1], many studies suggest SOD1 toxic gain of function through protein misfolding and aggregation, associated with aberrant cellular function [5,6]. Supporting this notion are reports that insoluble aggregates are SOD1-immunoreactive in both familial [7] and sporadic ALS cases [8], as well as in ALS mouse models [9,10], and that mutant SOD1 misfolding or aggregation is inversely correlated with disease duration [11,12]. Moreover, SOD1 mutants have been shown to interact with ER [13,14] and mitochondria [15,16,17], solely in nervous system tissues, and are specifically associated with mitochondrial dysfunction [16] and ER stress induction [14]. Furthermore, studies have demonstrated fibrillar, amyloid-like [5,18,19,20,21] filaments of misfolded SOD1 inclusions in human patients [22,23] and mouse models [24,25] of the disease.

Many strategies have been investigated as therapeutic targets for ALS and other neurodegenerative diseases. Among them are chemical chaperones, which have the potential to restore protein homeostasis and improve cellular function [26]. A well-known hydrophobic chemical chaperone [27], 4-phenylbutyric acid (4-PBA, Figure S1), was recently tested in ALS clinical trials in which 4-PBA combined with a taurursodiol treatment had both functional and survival benefits [28,29]. As a short-chain fatty acid 4-PBA is bioavailable as well as a blood-brain barrier (BBB)-permeable compound, which has already been approved by the Food and Drug Administration (FDA) to treat urea cycle disorders in children [27]. Even though the mechanism of action of 4-PBA has not been fully established yet, it is proposed that 4-PBA hydrophobic regions interact with exposed hydrophobic segments of unfolded proteins, thereby reducing protein aggregation levels [30]. Moreover, 4-PBA was also shown to have a potential therapeutic effect in several mouse models of other neurodegenerative disorders, also characterized by protein aggregation, such as Alzheimer’s disease [31] and Huntington’s disease [32]. Despite these accumulating findings, suggesting a positive effect of 4-PBA treatment, the main drawback to its therapeutic use is the high dosage required—0.5 to 20 g per kg per day [30], a nonrealistic dosage for treating chronic disorders such as ALS [33].

**Figure 1:**
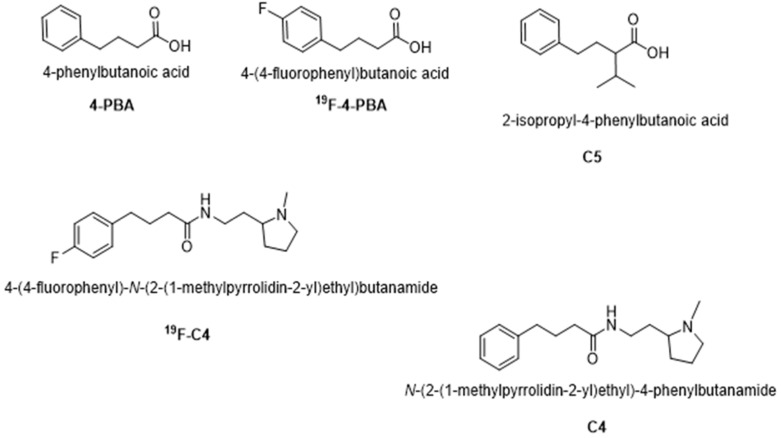
Molecular structures of 4-PBA, ^19^F-PBA, C4, ^19^F-C4, and C5.

Recently, Azoulay-Ginsburg S. and colleagues identified and synthesized 4-PBA derivatives in order to reduce the effective concentration required to achieve a therapeutic effect. Two 4-PBA derivatives, *N*-(2-(1-methylpyrrolidin-2-yl)ethyl)-4-phenylbutanamide (compound **4**, C4) and 2-isopropyl-4-phenylbutanoic acid (compound **5**, C5) (Figure 1), exhibited positive outcomes when tested in several in vitro and in vivo models of neurodegenerative diseases, characterized by protein aggregation [33,34]. More specifically, compared to 4-PBA, the identified chemical chaperone C5 was more effective in protecting cells under ER stress conditions in vitro. C5 also reduced the secretion rate of mutant Neuroligin3 (an autism-related protein), acted as a direct chemical chaperone for purified proteins, and reduced protein aggregation in the PC12 neuronal cell model [33]. The other chemical chaperone synthesized, C4, was selectively targeted to the lysosome, part of the cell clearance system, which may increase its effectivity and reduce its effective therapeutic concentration. Furthermore, C4 exhibited a protective effect on cell viability following ER stress in PC12 cells, and significantly reduced the retinal degeneration tested on a C9orf72 Drosophila melanogaster ALS model [34].

In the current study, we investigated the potential therapeutic effect of C4 and C5 in the mutant SOD1^G93A^ mouse model of ALS, combining both in vitro and in vivo studies. We hypothesized that C4 and C5 chaperone activity could reduce the levels of mutant SOD1 misfolding and/or aggregation, which may result in improved motor function as well as the prolonged survival rate of the transgenic mice. Although C4 and C5 successfully suppressed recombinant mutant SOD1^G93A^ amyloid aggregation levels in vitro, C4 or C5 daily injections to adult mice commencing after disease onset did not significantly affect disease progression or survival of the treated SOD1^G93A^ mice.

## 2. Results

### 2.1. C4 and C5 Chemical Chaperones Strongly Inhibited the Formation of SOD1 Amyloid Aggregation In Vitro 

**Figure 2:**
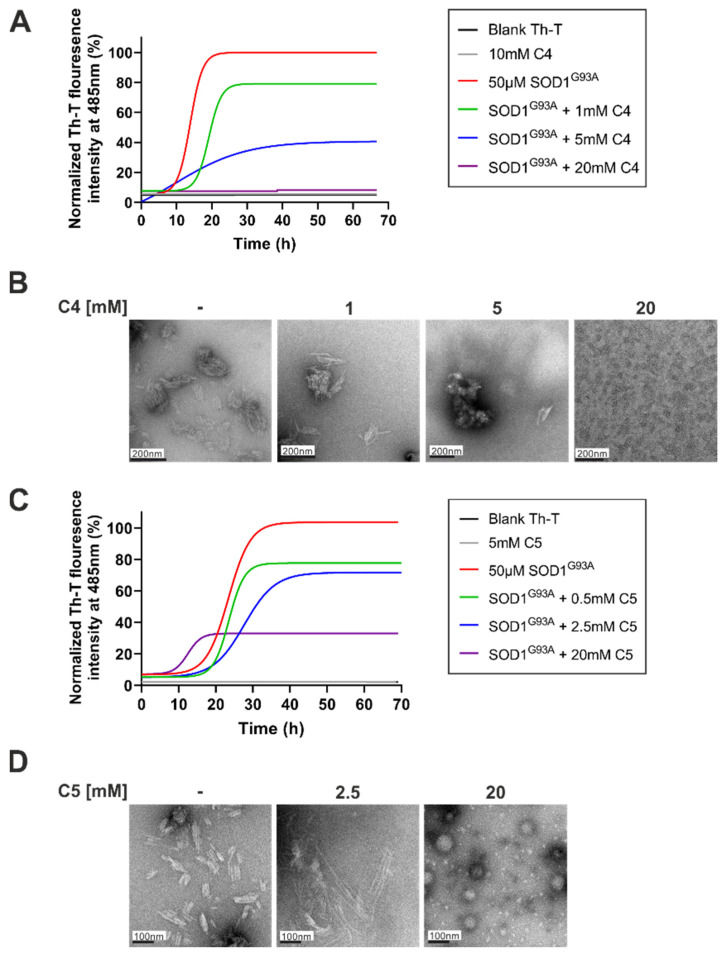
C4 and C5 strongly inhibited the formation of SOD1^G93A^ amyloid aggregation in vitro. (**A**,**C**) Recombinant SOD1^G93A^ amyloid aggregation was determined by monitoring Th-T fluorescence intensity during co-incubation of SOD1^G93A^ solution (50 µM) with different molar ratios of C4 (1–20 mM, (**A**)) or C5 (0.5–20 mM, (**C**)) at 37 °C for ~70 h. Fluorescence was normalized to the maximal Th-T fluorescence intensity elucidated by SOD1^G93A^ alone. Data points represent the average results from one representative experiment (performed in triplicates) of three independent experiments. Fluorescence was fitted to the Boltzmann sigmoidal equation using GraphPad Prism software. (**B**,**D**) TEM images of SOD1^G93A^ solution (50 µM) after 70 h incubation at 37 °C alone or with the presence of 1–20 mM of C4 (**B**) or 0.5–20 mM of C5 (**D**).

C4 and C5 chemical chaperones are derivatives of 4-PBA (Figure 1) and were shown previously to inhibit protein aggregation both in vitro and in vivo in models of autism spectrum disorder (ASD) and C9orf72-related ALS [33,34]. In order to determine the effect of C4 and C5 on SOD1^G93A^ aggregation, we purified and incubated recombinant SOD1^G93A^ protein under aggregation-promoting conditions (i.e., in the presence of a reducing agent and a metal chelator), with increasing concentrations of C4 (1–20 mM) (Figure 2A) and C5 (0.5–20 mM) (Figure 2C). We measured the kinetics of amyloid aggregation by monitoring the fluorescence intensity signal of thioflavin-T (Th-T) at 485 nm over a period of about 70 h. Incubating SOD1^G93A^ alone resulted in an exponential increase in Th-T fluorescence intensity, indicating formation of amyloid fibrillar aggregates (Figure 2A,C). This was confirmed by transmission electron microscopy (TEM) imaging performed at the end of the incubation period (after 72 h), which revealed a fibrous aggregated formation (Figure 2B,D). In contrast, incubating SOD1^G93A^ in the presence of C4 (Figure 2A,B) or C5 (Figure 2C,D) reduced the fluorescent signal in a dose-dependent manner, suppressed the formation of fibrillar aggregates, and switched the aggregation pattern to an amorphous disordered one (Figure 2B,D). The fluorescent signal measured during the incubation of C4 or C5 alone without recombinant SOD1 was undetectable in the Th-T analysis, indicating that they did not produce aggregates whatsoever (Figure 2A,C).

### 2.2. Daily Injection of C4 or C5 Chemical Chaperones Following Disease Onset Had No Effect on Disease Progression and Survival of Mutant SOD1^G93A^ Mice

**Figure 3:**
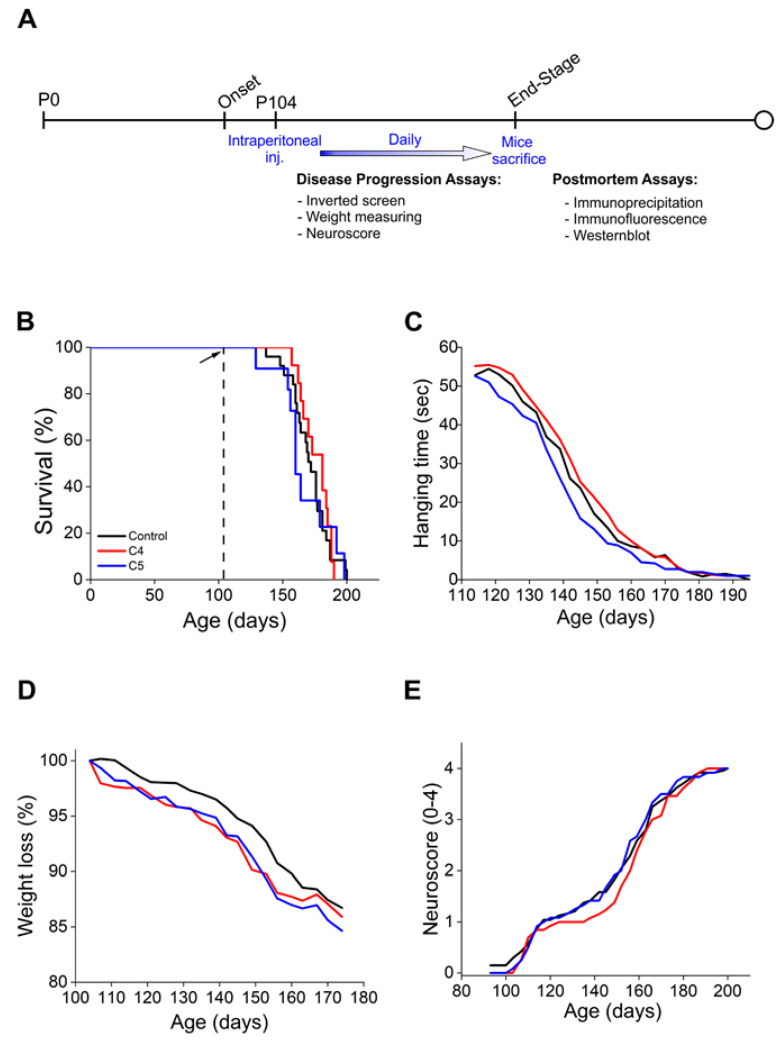
C4 and C5 treatment did not significantly extend the survival or improve the motor symptoms of SOD1^G93A^ mice. (**A**) Schematic representation of the experimental design. Treated SOD1^G93A^ mice received daily intraperitoneal injections of C4 (red, *n* = 12) or C5 (blue, *n* = 12) starting at p104. The control group includes noninjected and acetate-buffer-injected mice (black, *n* = 24). (**B**) C4 and C5 treatment does not significantly extend the survival of SOD1^G93A^ mice. (**C**) Motor function of C4- and C5-treated mice and control mice evaluated by the inverted screen test. (**D**,**E**) Disease progression of C4- and C5-treated, and control mice evaluated by weight loss rate (**D**) and NeuroScore (**E**).

To test the potential therapeutic effect of C4 and C5 in a mouse model of ALS, we used the most studied mutant SOD1^G93A^ model. We alleged that C5, as a α-isopropyl derivative of 4-PBA, would pass the BBB, as is the case with 4-PBA [35]. In order to determine whether C4 is indeed able to cross the BBB, its fluorinated derivative was synthesized as described [33]. Wild type mice were intraperitoneally (IP) injected with 50 mg of ^19^F-labeled C4 in acetate buffer, and 30 min later the brains were harvested and analyzed by nuclear magnetic resonance (^19^F-NMR). Two clear ^19^F-NMR signals were observed in injected but not in the noninjected mouse brain tissue (Figure S1A,B). Two ^19^F-NMR peaks in −118.03 ppm and −118.26 ppm were obtained: one related to the ^19^F-labeled 4-phenylbutyric acid (Figure 1) and the other to ^19^F-C4. We assume that the compound passed the BBB and was partially hydrolyzed (Figure S1C). Then, based on this positive penetration outcome, transgenic SOD1^G93A^ mice (males and females) were IP injected daily, starting at postnatal day 104 (following disease onset), with either C4 (100 mg/kg, in acetate buffer), C5 (25 mg/kg, in PBS), or 200 µL acetate buffer (pH 5.6) as a control. Disease progression was monitored until the end stage by the neurological scoring system NeuroScore (NS), the inverted screen test, and the routinely used mice weight loss rate (Figure 3A). C4 or C5 daily injections starting after disease onset did not significantly extend SOD1^G93A^ mice survival (Figure 3B; Control: 171 d; C4: 176 d; C5: 165 d). Moreover, the treatment did not significantly affect either the mice clinical neuroscore (Figure 3E) or their weight loss rate (Figure 3D) and did not improve the mice motor function as analyzed by the inverted screen test (Figure 3C). Since gender differences exist in this transgenic model, the males and females were also analyzed separately. Confirming our previous results, we were not able to detect any statistical effect on survival, neither in males nor in females (Figure S2).

### 2.3. C4 or C5 Treatment Starting after Disease Onset Failed to Rescue the Motor Neurons and Reduce the Activation of Astrocytes and Microglia in the Spinal Cord of SOD1^G93A^ Mice

**Figure 4:**
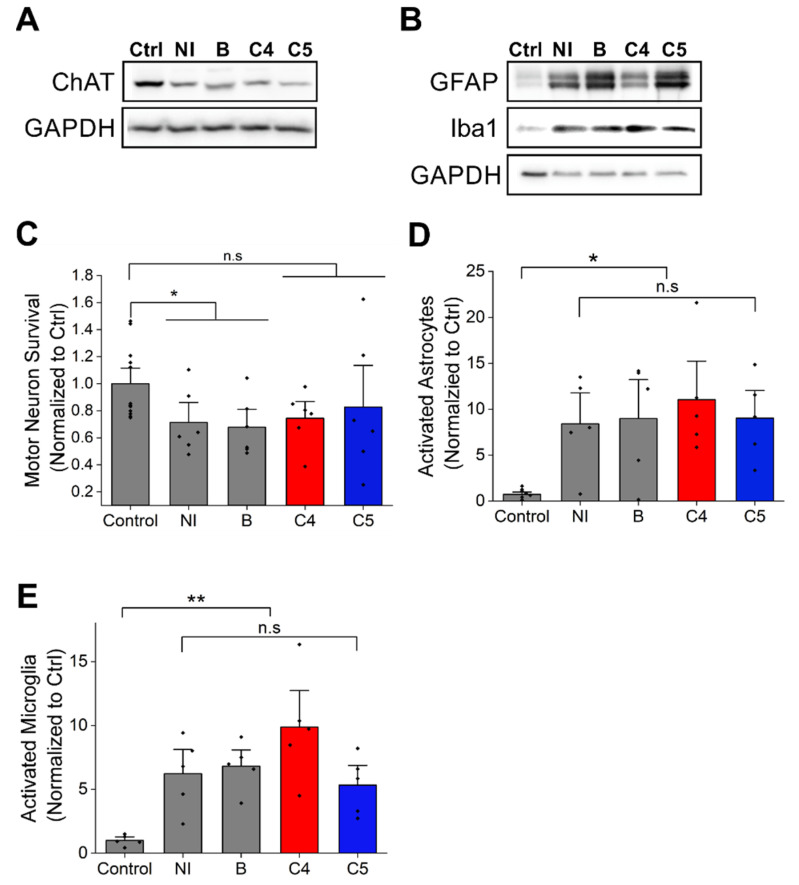
C4 and C5 treatment had no effect on motor neuron survival or the neuroinflammatory response in SOD1^G93A^ mice spinal cords. (**A**) Motor neurons survival was evaluated by immunoblot of lysates from spinal cords of non-transgenic and SOD1^G93A^ mice with anti-ChAT antibody. (**B**) Quantification of ChAT level intensity in non-transgenic mice (control) and C4- and C5-treated and untreated SOD1^G93A^ mice. (**C**–**E**) Neuroinflammatory response was evaluated by immunoblot of lysates from spinal cords of non-transgenic and SOD1^G93A^ mice with anti-GFAP and anti-Iba1 antibodies (**C**). (**D**) Quantification of activated astrocytes (GFAP antibody, D) and activated microglia (Iba1 antibody, (**E**)) in non-transgenic and SOD1^G93A^ mice untreated or treated with C4 or C5. Ctrl- non-transgenic mouse; NI- SOD1^G93A^ noninjected; B- SOD1^G93A^ injected with acetate buffer. Quantification analysis was performed with Student’s *t*-test. Bars represent mean ±  SEM. Data indicate one representative experiment out of 3–5 independent experiments. n.s, nonsignificant; * *p* < 0.05; ** *p* < 0.01.

In order to determine whether the 4-PBA derivatives have any effect on motor neuron survival, immunoblot against ChAT, a motor neuron marker was performed. ChAT expression levels were reduced in mutant SOD1^G93A^ spinal cord compared to littermate non-transgenic controls (Figure 4A), with daily injections of C4 or C5 starting after onset having no effect on motor neuron survival (Figure 4A,C). Moreover, ALS pathology is accompanied by non-cell autonomous processes [36,37]. Specifically, studies report widespread activation as well as impaired function of astrocytes [9,38] and microglia in both ALS patients and mouse models [39]. Indeed, immunoblotting of spinal cords of mutant SOD1^G93A^ mice showed a strong increase in astrocyte (GFAP) and microglia (Iba1) activation (Figure 4B,D,E) in their spinal cord with no difference in this activation pattern (Figure 4B,D,E) following C4 or C5 administration compared with untreated mice. These results were confirmed by immunofluorescence of untreated and treated mutant SOD1^G93A^ spinal cord sections (Figure S3).

### 2.4. Daily Injection of C4 or C5 Chemical Chaperones Starting after Disease Onset Failed to Reduce the Accumulation of Misfolded SOD1

**Figure 5:**
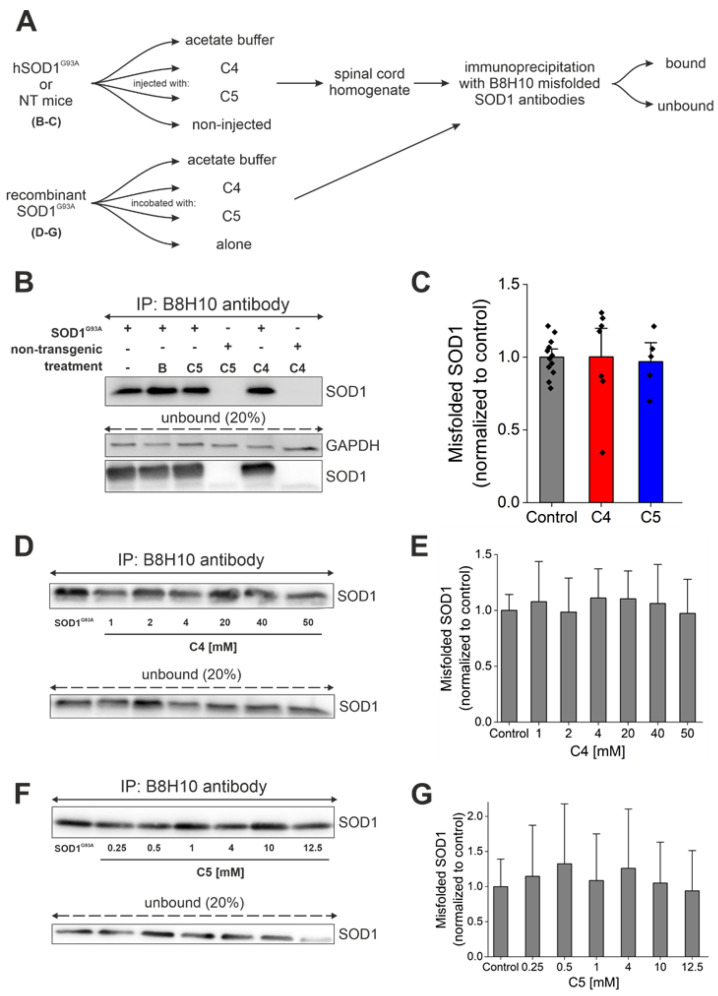
C4 or C5 chemical chaperones did not reduce SOD1 misfolding in vitro or in vivo. (**A**) Schematic representation of the experimental protocol. (**B**) Misfolded SOD1 levels were detected by immunoprecipitation produced with B8H10 antibody from mutant SOD1^G93A^ spinal cords. IP—immunoprecipitation; B—acetate buffer. (**C**) Quantification of misfolded SOD1 levels in untreated (*n* = 12, grey) and C4 (*n* = 7, red) or C5 (*n* = 5, blue)-treated mutant SOD1^G93A^ spinal cords. (**D**,**F**) Misfolded SOD1 levels were detected by immunoprecipitation with B8H10 antibody after incubating recombinant SOD1^G93A^ protein (2 µg) with different concentrations of C4 (1–50 mM) (**D**) or C5 (0.25–12.5 mM) (**F**). (**E**,**G**) Quantification of misfolded SOD1 levels of mutant SOD1^G93A^ protein without or with C4 (**E**) or C5 (**G**) incubation. Quantification analysis was performed with Student’s *t*-test. Bars represent mean ± SEM. Data indicate one representative experiment out of 3–5 independent experiments.

Immunoprecipitation with a conformational antibody that specifically recognizes misfolded SOD1, B8H10 [40,41], was used to determine the accumulation of misfolded SOD1 in spinal cords of treated and untreated SOD1^G93A^ mice (Figure 5A). Mutant SOD1^G93A^ mice injected with C4 or C5 had similar accumulation of misfolded SOD1 in the lumbar spinal cord (Figure 5B,C) and in the brain (Figure S4) compared with that of SOD1^G93A^ mice injected with acetate buffer as control (bound fraction). 

Supporting these findings, end-stage mice were perfused with 4% PFA, and frozen sections of spinal cord were immunostained by the free-floating technique with the same conformational antibody against misfolded SOD1, B8H10, to compare the accumulation of misfolded SOD1 in the spinal cords of treated and nontreated SOD1^G93A^ mice (Figure S5). Misfolded SOD1 accumulation levels were not significantly reduced in the spinal cord of C4- (Figure S5A) or C5 (Figure S5B)-injected mice compared to nontreated SOD1^G93A^ mice. Moreover, we have tested the ability of C4 (Figure 5D,E) and C5 (Figure 5F,G) to reduce misfolded SOD1 accumulation of recombinant SOD1^G93A^ protein. C4 and C5 were incubated with recombinant SOD1^G93A^ at different concentrations (C4, 1–50 mM; C5, 0.25–12.5 mM). Immunoprecipitation produced by the B8H10 antibody showed no difference in the accumulation of misfolded SOD1 following incubation with C4 (Figure 5D,E) or C5 (Figure 5F,G).

### 2.5. Daily Injection of C4 or C5 Chemical Chaperones Following Disease Onset Failed to Reduce Total Aggregate Formation of Mutant SOD1

**Figure 6:**
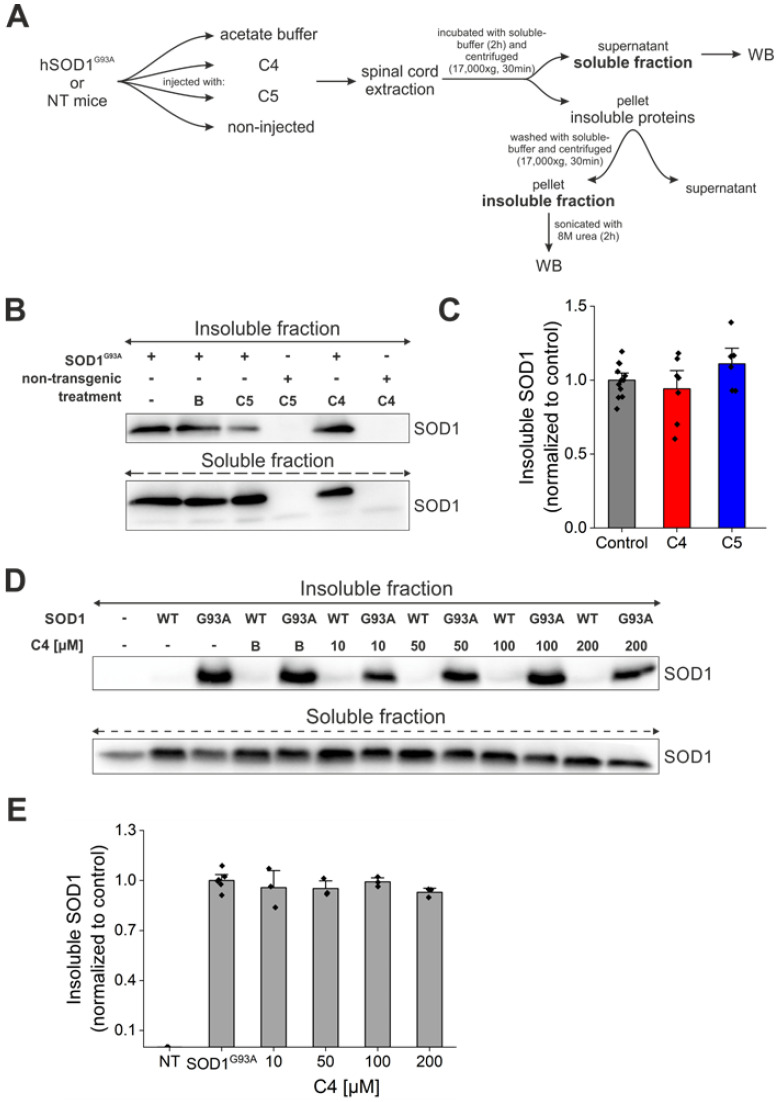
C4 or C5 chemical chaperones did not reduce SOD1 aggregate formation in vivo. (**A**) A schematic representation of the experimental protocol. (**B**) SOD1 aggregation levels were detected by immunoblotting of soluble and SDS-insoluble fractions of spinal cords from untreated and C4- or C5-treated mutant SOD1^G93A^ mice. Anti-GAPDH antibody was used to detect total protein loading amounts. Data indicate one membrane out of 3 independent experiments. (**C**) Quantification of the band intensity representing the insoluble SOD1 in fractions of spinal cords from untreated (*n* = 12, grey) and C4 (*n* = 7, red) or C5 (*n* = 6, blue) treated mutant SOD1^G93A^ mice performed with Evolution-Capt Edge software. (**D**,**E**) SH-SY5Y neuronal cells were transfected with hSOD1^WT^ or hSOD1^G93A^ plasmids and treated with different concentrations of C4 (10–200 µM). (**D**) SOD1 aggregation levels were detected by immunoblotting of insoluble fractions of the cell lysates. (**E**) Quantification of bands intensity of SOD1 insoluble to total SOD1 amount (soluble + insoluble). NT—non-transfected. Quantification analysis of the relative aggregation propensity performed with student t-test. Bars represent mean ± SEM.

In order to determine whether C4 or C5 had any effect on total protein aggregation in our treated mice, we separated the soluble and insoluble fractions from the spinal cords of the treated and nontreated mutant SOD1^G93A^ mice (Figure 6A). Our analysis revealed no significant difference in SOD1 aggregation levels in the C4- or C5-injected mice compared to the untreated SOD1^G93A^ mice (Figure 6B,C). In addition, we transfected SH-SY5Y neuronal cells with SOD1^WT^ and SOD1^G93A^ plasmids, followed by incubation with C4 at increasing concentrations (10–200 µM). We separated the soluble and insoluble fractions 72 h post-transfection and compared SOD1 aggregation levels in the insoluble fraction. Immunoblotting revealed no difference in SOD1^G93A^ aggregation levels following incubation with C4 at the tested concentrations (Figure 6D,E). 

### 2.6. High Levels of C4 and C5 Reached the Brain and Spinal Cord for a Short Period of Time

Finally, in order to determine whether sufficient amounts of both tested compounds indeed reach the brain and spinal cord, the classical PK experiment (single dose injection) was conducted, as described in “Materials and Methods”. The obtained t_1/2_ of tested compounds showed that C4 t_1/2_ and C5 t_1/2_ in serum were approximately 48 min and 20 min, respectively (Figure S6A,D). The maximal amount of C4 in the brain was detected after 30 min: 0.189 ± 0.02 mM and after 3 h the concentration of C4 in the brain had dropped by 10-fold (Figure S6B). Moreover, in the spinal cord after 30 min, the compound reached its peak concentration: 0.338 ± 0.02 mM, and similarly to the brain, by 3 h C4 concentration decreased by 10-fold (Figure S6C). C5 reached the highest concentration in the brain immediately following administration (approximately after 6 min): 0.175 ± 0.043 mM, and by 30 min, the amount of the compound in the brain was negligible (Figure S6E). Moreover, in the spinal cord, the compound showed a similar pattern of behavior: 0.136 ± 0.024 mM in the beginning, and after 30 min, only a minimal concentration of the compound was detected (17-fold lower compared to the starting level) (Figure S6F).

## 3. Discussion

In this study, we evaluated the potential therapeutic effect of the chemical chaperones C4 and C5, two 4-PBA derivatives which exhibited positive outcomes when tested in several models of protein aggregation diseases [33,34], on the mutant SOD1^G93A^ model of ALS. We found that C4 and C5 strongly inhibited the formation of mutant SOD1^G93A^ amyloid aggregates in a dose-dependent manner in vitro by changing the aggregation pattern from amyloid aggregation to an amorphous less toxic one [42], as examined by the Th-T assay and confirmed by TEM imaging. However, daily injections of C4 or C5 into a SOD1^G93A^ mouse model, starting after disease onset, failed to significantly affect disease progression or survival of treated mice.

Correlating with these in vivo findings, was the observation that the accumulation of soluble misfolded SOD1 was not reduced in the spinal cord and brain of C4- and C5-injected mice. Moreover, SOD1 aggregation in end-stage spinal cords was only slightly (but not significantly) reduced by C4 and C5 daily injections, as revealed by a soluble–insoluble assay and confirmed by immunofluorescence staining. Finally, our treatment had no effect on the neuroinflammatory response in SOD1^G93A^ mice lumbar spinal cord. 

A key question in the field of protein misfolding-related disorders is whether the toxic species is the soluble misfolded form of the protein or the formation of insoluble cytoplasmic inclusions. Specifically, the benefits and harmful processes associated with the formation of insoluble aggregates are still being investigated [43]. Traditionally, the presence of protein inclusions in neurodegenerative diseases has been related to the cell’s failure to refold misfolded proteins by chaperone activity [44]. However, other findings highlight the possibility that inclusion formation is not necessarily pathological [45]. Amyloid fibril formation is an intrinsic property of proteins in general [46,47], and there is accumulating evidence that this property may serve as a protective response, essential for several biological activities [45,48]. For example, protein aggregation into insoluble deposits was reported as a protective mechanism against oxidative stress [44] to allow efficient cell cycle restart after stress [49]. Recently, SOD1 insoluble aggregate formation was suggested as a protective mechanism to reduce the amount of toxic SOD1 trimers [50,51]. In addition, it was shown that inclusion body formation can function as a coping response to toxic mutant huntingtin [52]. Moreover, it was suggested that the aggregation process itself is related to toxicity, and that a common mechanism of toxicity is involved in several aggregation-related disorders [43]. In light of these findings, we raise the question of whether reducing the solubility of mutant proteins through aggregation may be part of the cell’s protective strategy, and whether the soluble misfolded SOD1 form is indeed the most toxic. Since the inhibition of SOD1 amyloid aggregation observed in vitro could not be replicated in our treated mice, we cannot make any definitive claim on this issue. 

In an attempt to explain the lack of in vivo effect of the compounds on SOD1^G93A^ pathogenesis, it is worth considering the possibility that C4 or C5 injection starting at an earlier time point, prior to disease onset, might have been more beneficial. The 4-PBA was already tested in the SOD1^G93A^ ALS mouse model [53,54,55]. These studies showed that treating mice prior to manifestation of clinical symptoms resulted in extended survival rate, in addition to improved body weight loss rate as well as improved motor function [53,55]. Supporting this notion is our in vitro Th-T assay, where C4 and C5 suppressed SOD1 amyloid aggregation before its formation. Starting with C4 or C5 injections at the presymptomatic stage would likely suppress the formation of amyloid aggregates and thus potentially affect disease progression. 

We hypothesized that, when compared to 4-PBA, C4 and C5 may be effective in inhibition of aggregation at lower, more therapeutically relevant, concentrations. However, our Th-T results, accompanied by TEM imaging, revealed a complete suppression of SOD1^G93A^ amyloid aggregates during incubation with relatively high concentrations of the compounds, suggesting that the synthesis of these new 4-PBA derivatives failed to achieve therapeutic outcomes at low dosages, as we had expected. Supporting these findings was a soluble–insoluble assay, where the incubation of SOD1^G93A^-expressing human neuronal cells with lower concentrations of C4 failed to reduce SOD1 aggregation levels.

Furthermore, previous findings from SOD1^G93A^ mice show that an effective therapeutic dosage was achieved by the administration of 200–400 mg/kg per day of 4-PBA [53,54,55]. Here, we aimed to test whether the new synthesized 4-PBA derivatives would present positive outcomes when administrating significantly lower dosages (100 mg/kg per day for C4 and 25 mg/kg per day for C5). Important to mention is that, even such high doses of both compounds that resulted in very high concentrations in the brain and spinal cord (mM range), although for a very short time, did not provide the expected outcome on disease progression and survival of SOD1^G93A^. Thus, increasing C4 and C5 stability might have resulted in slower disease progression.

In addition, we tested the potential therapeutic effect of C4 and C5 on the early onset and very aggressive SOD1^G93A^ model of ALS [56]. Other SOD1 ALS-related mutations might present different outcomes. Likewise, C4 or C5 may act as chaperones for other ALS-related protein aggregates, such as ALS models presenting cytosolic TDP-43 inclusions, which may also involve other pathological mechanisms.

Riluzole [57,58] and edaravone [57,59] are the only two drugs approved by the FDA as therapeutic agents in ALS to date; however, their mechanism of action is not fully understood. Riluzole, a glutamate antagonist, appears to reduce damage to MNs by averting excitotoxicity [60,61]. Although riluzole was approved in 1995 based on clinical trials [58,62], more recent studies testing its therapeutic effect in currently available ALS-relevant mouse models revealed that it failed to improve the lifespan of the treated mice [63]. Edaravone is an antioxidant with a free radical-scavenging activity which successfully reduced oxidative stress and improved the motor performance of SOD1^G93A^ mice [59,64,65,66]. Both riluzole and edaravone are mildly effective, prolonging some patients’ survival by up to 2–3 months, and have a beneficial effect only when taken at the first few months after diagnosis [67]. Combining 4-PBA treatment with riluzole [55] or with the antioxidant AEOL 10150 [54] was reported to improve disease outcome and extended mice survival rate [54,55]. Thus, combining the chaperone activity of C4 or C5 with other known therapeutic strategies might be more effective in eliminating mutant SOD1 toxicity. 

In conclusion, our findings suggest that the use of such chemical chaperones alone may not be realistic due to their high and barely tolerable active doses and bad PK parameters. Thus, the chemical chaperone-based strategy for ALS drug development may need to be optimized for more effective results.

## 4. Materials and Methods

### 4.1. Chemical Chaperones Synthesis

C4 and its fluorinated derivative ^19^F-C4 were synthesized as described [34]. C5 was purchased from Enamine, Kyiv, Ukraine.

### 4.2. Animals and Injection Protocol

Altogether, 48 B6 background (C57BL/6J TgN-SOD1-G93A-Gur; SOD1^G93A^) female and male mice were used for the experiments. The treatment protocol was approved by the Animal Care and Use Committee of Ben-Gurion University of the Negev, as required by Israeli legislation. Mice received 200 µL final volume of intraperitoneal (IP) injections every 24 h. C4-treated mice received 100 mg/kg/day diluted in acetate buffer, pH 5.6. C5-treated mice received 25 mg/kg/day diluted in PBS, pH 7.3. The control group included noninjected mice and mice injected only with acetate buffer.

All behavioral tests and body weight measurements were conducted twice a week. The inverted screen test [68] and the neurological score [69] were assessed as described previously.

### 4.3. ^19^F-NMR

A solution of compound 4 (labeled by the fluorine atom: ^19^F-C4), Figure S1 (50 mg, final concertation: 0.34 M), was prepared in acetate buffer, pH = 5.6. The acidic pH of the formulation was used to ensure the solubility of the compound as a quaternary ammonium salt in the blood. Thirty minutes post-IP administration of the compound, mice were euthanized, the brain was reperfused by saline, isolated, and homogenized. The entire brain homogenate was mixed with D_2_O and the ^19^F-NMR analysis was conducted as described [34].

### 4.4. SOD1^G93A^ Protein Purification

Recombinant mutant SOD1^G93A^ was purified as previously described [70]. Briefly, sequence of human SOD1^G93A^ was optimized for codon usage in *E. Coli*, cloned into pHIS1 vector [71], and expressed as 6HIS-tagged (N-term) soluble protein in BL21 (New England BioLabs, Ipswich, MA, USA) cells. An amount of 0.5 mL of bacteria culture were grown in 100 mL enriched lysogenic broth (LB) medium (2% tryptone, 1% yeast extract, 0.5% NaCl) at 37 °C for 3 h. Grown culture was added to new enriched LB (2% tryptone, 1% yeast extract, 0.5% NaCl, 0.2% sterile glycerol) containers, and the cultures were grown at 30 °C until turbidity at 600 nm reached 0.6–0.8 optical density (OD). The expression of the protein was induced by the addition of 0.1 mM of isopropyl β-D-1-thiogalactopyranoside (IPTG), followed by overnight incubation at 20 °C. Bacteria cells were harvested by 30 min centrifugation (7434.64× *g*) at 4 °C, the pellet was washed in 50 mM Tris-HCl buffer (pH 8), followed by an additional 20 min of centrifugation. After 30 min incubation on ice in sonication buffer (0.5 M NaCl, 50 mM Tris-HCl pH 7.5, 10 mM imidazole, 2 mM β-mercaptoethanol, 0.1 mM PMSF, 1 µL/mL protease inhibitor (PI, APExBio), 1 mg/mL lysozyme), the cells were disrupted by sonication for 3 min (amplitude: 95%, 20 s on and 40 s off; 18 °C). To remove the DNA, the crude extract was incubated on ice for 30 min in the presence of 25 µg/mL bovine pancreas DNaseI (Merck, Israel) and 5 mM MgSO_4_, followed by 30 min centrifugation (1486× *g*) at 4 °C. The supernatant was loaded on a 5 mL HisTrap FF column (Cytiva, Marlborough, MA, USA), using ÄKTA pure protein purification system (GE Healthcare, Chicago, IL, USA) equilibrated with binding buffer (0.5 M NaCl, 50 mM Tris-HCl pH 7.5, 10 mM imidazole, 2 mM β-mercaptoethanol). The column was washed with washing buffer (0.5 M NaCl, 50 mM Tris-HCl pH 7.5, 20 mM imidazole, 2 mM β-mercaptoethanol), and the protein was eluted by a linear 20–400 mM imidazole gradient. The peak fractions were dialyzed (8–12 h each) at 4 °C against dialysis buffer 1 (10 mM EDTA, 100 mM sodium acetate, pH 3.8), followed by dialysis against dialysis buffer 2 (0.1 M NaCl, 100 mM sodium acetate, pH 3.8) three times, followed by dialysis against dialysis buffer 3 (0.1 M NaCl, 100 mM Sodium Acetate, pH 5.5, 10% glycerol) twice. Afterwards, the fraction was centrifuged at 100,000× *g* at 4 °C for 1 h using ultracentrifuge (Sorvall M120, Discovery, AZ, USA), and the supernatant was stored at −80 °C until use. Protein concentration was measured by the Bradford method using bovine serum albumin as standard.

### 4.5. Thioflavin-T (Th-T) Aggregation Assay

Recombinant mutant SOD1^G93A^ protein (50 µM) with or without C4 (10–50 mM) was incubated in 200 µL HEPES buffer (HEPES bufferX4: 200 mM, NaCl 400 mMm (pH 7.4)), 5 mM Ethylenediaminetetraacetic acid (EDTA, Sigma, Burlington, MA, USA), and 1 mM Tris(2-carboxyethyl)phosphine hydrochloride (TCEP HCl, Sigma, Burlington, MA, USA) in the presence of 2 mM thioflavin T (Sigma, Burlington, MA, USA) in a black 96-well plate at 37 °C with fast continuous shaking. All samples were performed in triplicates and the fluorescence (λ_Ex_. = 440 nm; λ_Em_. = 485 nm) was measured at 15 min intervals for 70–80 h by Infinite M200 pro (Tecan, Mannedorf, Zurich, Switzerland).

### 4.6. Transmission Electron Microscopy (TEM)

TEM imaging was performed by the Nano-Fabrication Center team at Ben-Gurion University of the Negev, as described previously [21]. Briefly, at the end of the ThT aggregation assay (after ~70 h), 2.5 µL samples were deposited on a carbon-coated copper 300 grid. After 1 min, the excess liquid was carefully blotted onto filter paper, which was then dried at ambient temperature for 1 min. Uranyl acetate (5 µL, 2%) was added to the grid, and after 1 min, the excess of the salt solution was carefully removed with a filter paper. The imaging was performed using a ThermoFisher Scientific (FEI, Waltham, MA, USA) Talos F200C transmission electron microscope operating at 200 kV. The images were taken with Ceta 16M CMOS camera at various magnifications (100–500 nm), depending on the size of the fibril aggregates. The visible features were sensitive to the electron bean exposure, indicating their organic origin.

### 4.7. Cell Culture and Transfection

SH-SY5Y cells were maintained at 37 °C in a humidified, 5% CO_2_ incubator in Dulbecco’s modified Eagle medium (DMEM), supplemented with 10% tetracycline-free fetal bovine serum (FBS), 2 mM L-Glutamine, penicillin (100 units/mL), and streptomycin (0.1 mg/mL). Cells were split every two or three days (when they reached ~90% confluency) using Trypsin (Trypsin EDTA solution B 0.25%, EDTA 0.05%, Biological Industries, CAT# 03–052-1A). 

Cells were transfected using TurboFect^TM^ transfection reagent (Thermo, Waltham, Massachusetts, USA) according to manufacturer’s protocol. Briefly, cells were seeded ~0.8 × 10^5^ in 2 mL media (DMEM, 10% FBS, 2 mM L-glutamine, 100 units/mL ampicillin and 0.1 mg/mL streptomycin) in a 60 mm petri dish. Keeping a ratio of DNA:TurboFect^TM^ (1:2), 3 µg of DNA and 6 µL TurboFect^TM^ were dissolved in 200 µL of DMEM, mixed by vortex, and incubated for 25 min at room temperature. The mixture was added dropwise to the preseeded cells and incubated for 48 h at 37 °C in a humidified, 5% CO_2_ incubator.

### 4.8. Cell Lysis and Protein Extraction

Protocol was performed on ice. Briefly, media was removed, and cells were washed twice with 0.1 M PBS, and then lysed in 1 mL of ice-cold soluble buffer (0.1 M PBS, 1% Triton X, 5 mM EDTA, 10% Glycerol, 1 mM PMSF, 0.5% PI) with 20 min incubation at 4 °C. Cells were then detached using a cell scraper, collected into an Eppendorf, homogenized at 4000 RPM for 30 s, and centrifuged at 17,000× *g* for 30 min at 4 °C. The supernatant (soluble fraction) was collected and stored at −20 °C until use. The pellet was resuspended in 1 mL ice-cold soluble buffer (0.1 M PBS, 1% Triton X, 5 mM EDTA, 10% Glycerol) and centrifuged at 17,000× *g* at 4 °C for 30 min. Next, the pellet (insoluble fraction) was resolved using 400 µL of 8 M urea and sonication for 1 h at 4 °C. Protein concentration of soluble fractions was measured by the Bradford method using bovine serum albumin as standard, and the protein concentration of insoluble fractions was measured by the protein determination (BCA) kit (Cayman Chemical).

### 4.9. Tissue Harvesting and Protein Extraction

Brain/spinal cord (SC) tissues were dissected out, cut in half, and homogenized on ice in 3 volumes of ice-cold homogenization buffer (150 mM NaCl, 20 mM Tris-HCl pH 7.5, 1 mM PMSF, 1% triton, 1% PI (APExBio, Houston, TX, USA), 0.5% sodium deoxycholate, 0.1% SDS). Homogenates were centrifuged at 5000× *g* at 4 °C for 30 min, the supernatant (cytosolic fraction) was collected, and then stored at −80 °C until use. Protein concentration was measured by the Bradford method using bovine serum albumin as standard.

### 4.10. Immunoprecipitation (IP) Assay

Brain/SC tissue extracts (100 µg), or recombinant SOD1^G93A^ incubated with C4 (1–50 mM) or C5 (0.25–12.5 mM) for 1 h at 37 °C, were solubilized in IP buffer (0.5 M NaCl, 50 mM Tris (pH 7.4), 0.5% Nonidet P-40) and incubated at 4 °C overnight with B8H10 antibody (MediMabs, Montreal, Quebec), previously crosslinked to Dynabeads^TM^ protein G (Thermo, Waltham, MA, USA) according to the manufacturer’s instructions. The beads were magnetically isolated and, after crosslinking with the antibody, were washed three times with PBST (0.1 M PBS with 0.02% Tween20). After magnetic separation, unbound fractions were withdrawn for immunoblotting analysis. Beads were washed three times with IP buffer and the bound fractions were eluted by boiling for 5 min at 95 °C in X2 sample loading buffer.

### 4.11. Soluble-Insoluble Separation Assay

Spinal cord tissues were dissected out, cut in half, homogenized on ice in 200 µL of ice-cold soluble buffer (5 mM EDTA, 1 mM PMSF, 1% triton, 1% PI (APExBio, Houston, TX, USA), 0.1 M PBS), and incubated at 4 °C for 2 h while rotating. Homogenates were then centrifuged at 17,000× *g* at 4 °C for 30 min and the supernatant (soluble fraction) was collected and stored at −80 °C until use. The pellet was resuspended with 1 mL ice-cold soluble buffer (5 mM EDTA, 1% triton, 0.1 M PBS) and centrifuged at 17,000× *g* at 4 °C for 30 min. Next, the pellet (insoluble fraction) was resolved using 300 µL of 8 M urea and sonicated for 2 h at 4 °C. Protein concentration of soluble fractions was measured by the Bradford method using bovine serum albumin as standard, and protein concentration of insoluble fractions was measured by the protein determination (BCA) kit (Cayman Chemical).

### 4.12. Immunoblotting

Desired proteins were separated on a 10%, 12%, or 15% acrylamide SDS-PAGE gel in running buffer (25 mM Tris, 192 mM glycine, 0.1% SDS). Separated proteins were then blotted on a nitrocellulose membrane in transfer buffer (25 mM Tris, 192 mM glycine, 20% methanol). Blotted membranes were stained with ponceau-s (Sigma, Burlington, MA, USA) to verify protein presence and then washed several times with Tris-buffers saline with Tween20 (TBST) until color disappearance. The membranes were then incubated in 5% skimmed milk powder (Sigma, Burlington, MA, USA) in Tris-buffers saline (TBS) for 1 h while shaking to block exposed areas. The blocked membranes were incubated overnight at 4 °C while shaking with desired primary antibodies, including: mouse anti-SOD1 (1:1000, 24, SCB), mouse anti-GAPDH (1:200, SCB), rabbit anti-ChAT (1:1500, GeneTex), mouse anti-GFAP (1:1000, Merck), and rabbit anti-Iba1 (1:500, Abcam). The next day, the membranes were washed for 3 min in TBST followed by 3 × 3 min in TBS while shaking. Horseradish peroxidase (HRP)-conjugated goat anti-mouse or goat anti-rabbit IgG secondary antibodies (Jackson Immunochemicals, West Grove, PA, USA) were used and detected by the EZ-ECL reagent kit (Biological Industries, Kibbutz Beit-Haemek, Israel) containing the Luminol substrate for HRP. The membranes were photographed by Fusion Solo X (Vilber, Collegien, France). Reprobing of the membranes was performed in stripping buffer (Thermo, Waltham, MA, USA) for 15 min while shaking, followed by TBST and TBS washings.

### 4.13. Pharmacokinetic (PK) Study

A total of 26 mice (8–9 weeks old) were used in this study. The animals were randomly assigned to the treatment groups before the pharmacokinetic study. Six sampling time points (0, 0.5, 1, 3, 8, 24 h) were set for the experiment. Each of the time point treatment group included 3 animals. Test compounds: C4 (100 mg/kg) and C5 (25 mg/kg) were injected IP. Mice were sacrificed by cervical dislocation, and after that, the blood samples were collected by cardiac puncture, settled for 20 min, and then centrifuged for 10 min at 3000× *g* 4 °C degrees. Samples were snap-frozen and stored at −70 °C until subsequent analysis. An amount of 200 µL of acetonitrile was added to 50 µL of serum, and after centrifugation, the supernatant was used for mass spectroscopy analysis. Brains were harvested and lysed in 500 µL PBS using 18 G needle, and then 1 mL of acetonitrile was added. After the addition of the acetonitrile, the samples were homogenized again with the same needle. Spinal cords (SCs) were harvested and lysed in 250 µL PBS using 18 G needle, and then 500 µL of acetonitrile was added. After the addition of the acetonitrile, the samples were homogenized again with the needle. All procedures were conducted on ice. LC/MS from Agilent Technologies (Santa Clara, CA, USA) was used for PK analysis. Data were processed using mass L-ynX ver. 4.1 calculation and deconvolution software (Waters Corp., Milford, MA, USA). Spiking and calibration curve were generated using serum of the nontreated mice. 

### 4.14. Immunofluorescence

Mice were anesthetized via inhalation of 1 mL isoflurane, followed by perfusion with 50 mL 0.1 M PBS, and then switched to 4% paraformaldehyde (PFA) in 0.1 M PBS. The spinal cords were dissected out and postfixed in 4% formaldehyde in 0.1 M PBS at 4 °C overnight, cryoprotected in 20% sucrose in 0.1 M PBS 48 h at 4 °C, and afterwards embedded in Optimal Cutting Temperature (OCT) matrix compound (Tissue-Tek, Sakura Finetek). Sections were cut from the lumbar spinal cord (35 µm thickness), and the free-floating sections were stored in 0.1 M PBS with 0.02% sodium azide at 4 °C to conserve until staining. Sections were stained using standard protocol. First, sections were washed (3 × 15 min) in 0.1 M PBS with 0.03% Tween20 and then blocked for 1 h in blocking and permeabilization buffer (0.1 M PBS, 10% donkey serum, 1% BSA, 0.3% triton) with gentle agitation at room temperature. Sections were washed (3 × 15 min) in 0.1 M PBS with 0.03% Tween20 and then incubated overnight at 4 °C with gentle agitation, with primary antibodies made in 0.1 M PBS, 1% donkey serum, 1% BSA, 0.15% triton, including mouse anti-misfolded SOD1 (B8H10, 1:100, MediMabs, Montreal, Quebec), mouse anti-glial fibrillary acidic protein (GFAP, 1:400, Merck), goat anti-ionized calcium-binding adaptor molecule 1 (Iba1, 1:500, Abcam), and rabbit anti-neuronal nuclei antigen (NeuN, 1:200, Merck). The following day, the sections were washed (3 × 15 min) in 0.1 M PBS with 0.03% Tween20 and then incubated for 1 h at room temperature, gently shaking, with fluorescent conjugated secondary antibodies in 0.1 M PBS, 1% donkey serum, 1% BSA, 0.15% triton, including donkey anti-mouse (1:300, Alexa 555, Thermo), donkey anti-goat (1:200, Alexa 647, Abcam), donkey anti-mouse (1:200, Alexa 647, Abcam), and donkey anti-rabbit (1:200, Alexa 405, Abcam). Sections were then washed (3 × 15 min) in 0.1 M PBS and mounted on slides using Immu-Mount^TM^ mounting solution (Thermo), dried at room temperature overnight, and stored at 4 °C until imaging. Images were acquired on a NIKON C2Plus laser unit dock to a Nikon Eclipse *Ti* unit of the confocal microscope by using 10× and 20× objectives and 60× oil immersion objective. Scanning settings were reused across the samples.

### 4.15. Statistical Analysis

Quantification of band intensity across experiments was done using Evolution-Capt Edge software (version 18,08, Vilber, Collegien, France). The data was transferred and statistically analyzed using OriginPro software (2021, OriginLab, Northampton, MA, USA). Values are reported throughout as mean ± SEM. After confirming a normal distribution by the Shapiro–Wilk normality test, a one-way ANOVA was performed to compare the database between the 3 experimental groups. For nonlinear data, a Kruskal–Wallis test was performed instead. Significance was set at a confidence level of 0.05. 

ThT aggregation assay analysis was done using GraphPad Prism (La Jolla, CA, USA).

## Data Availability

Not applicable.

## Supplementary Materials

The supporting information can be downloaded at: https://www.mdpi.com/article/10.3390/ijms23169403/s1.
