# Supplementary material for: 4-Phenylbutyric Acid (4-PBA) Derivatives Prevent SOD1 Amyloid Aggregation In Vitro with No Effect on Disease Progression in SOD1-ALS Mice
Source: Int J Mol Sci. 2022 Aug 20;23(16):9403. doi: 10.3390/ijms23169403 (PMC9409193; doi:10.3390/ijms23169403)
Supplement: Supplementary file 1 [file ijms-23-09403-s001.zip › ijms-1845311-supplementary.pdf]

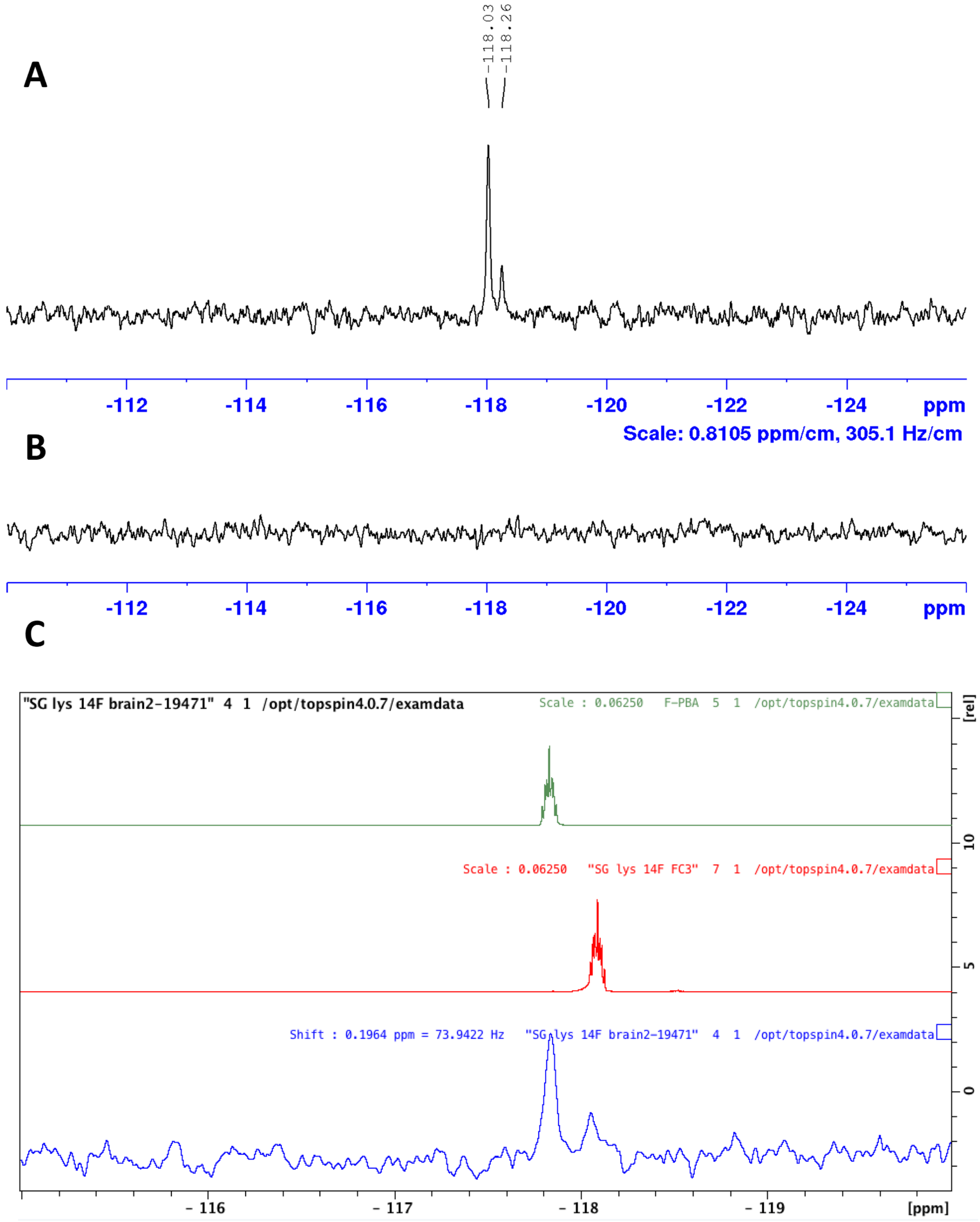

Figure S1: Detection of ^{19}F-NMR in brain homogenates from mouse treated with ^{19}F-C4 (**A**) or (**B**) treated with acetate buffer. (**C**) Comparison between ^{19}F-NMR spectra of ^{19}F4-PBA (green line), ^{19}F-C4 (red line) and the ^{19}F-NMR spectra from brain homogenate of mouse treated ^{19}F-C4 (blue line).

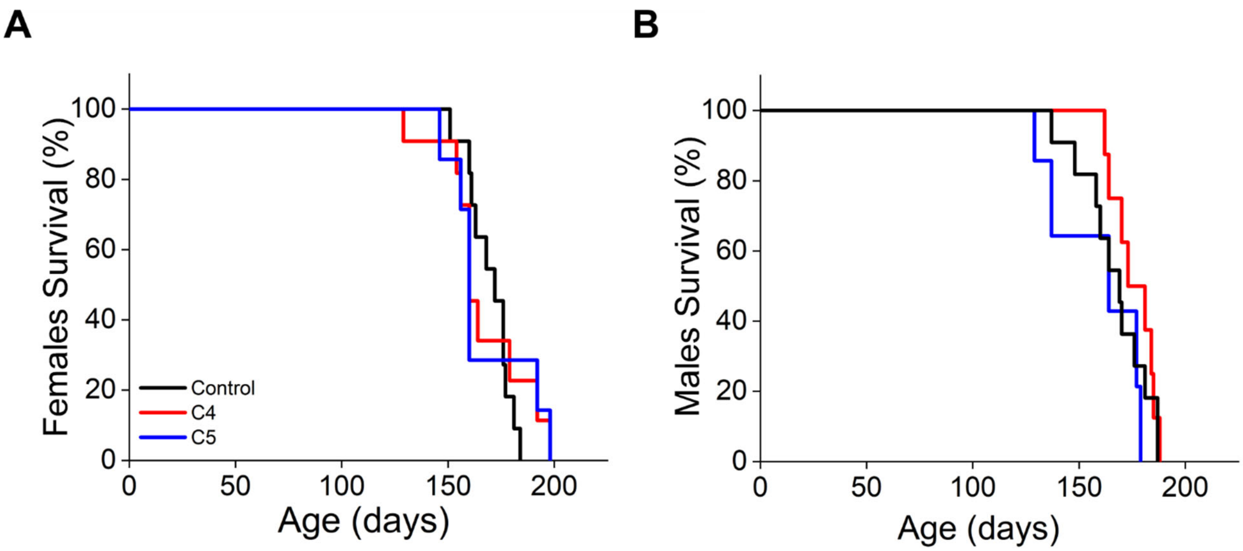

Figure S2: C4 and C5 treatment did not significantly extend the survival of SOD1^{G93A} mice. Treated SOD1^{G93A} mice received daily intraperitoneal injections of C4 (red, n=12) or C5 (blue, n=12), starting at p104. The control group includes non-injected and acetate buffer injected mice (black, n=24). **(A, B)** C4 and C5 treatment does not significantly extend the survival of females **(A)** and male **(B)** SOD1^{G93A} mice.

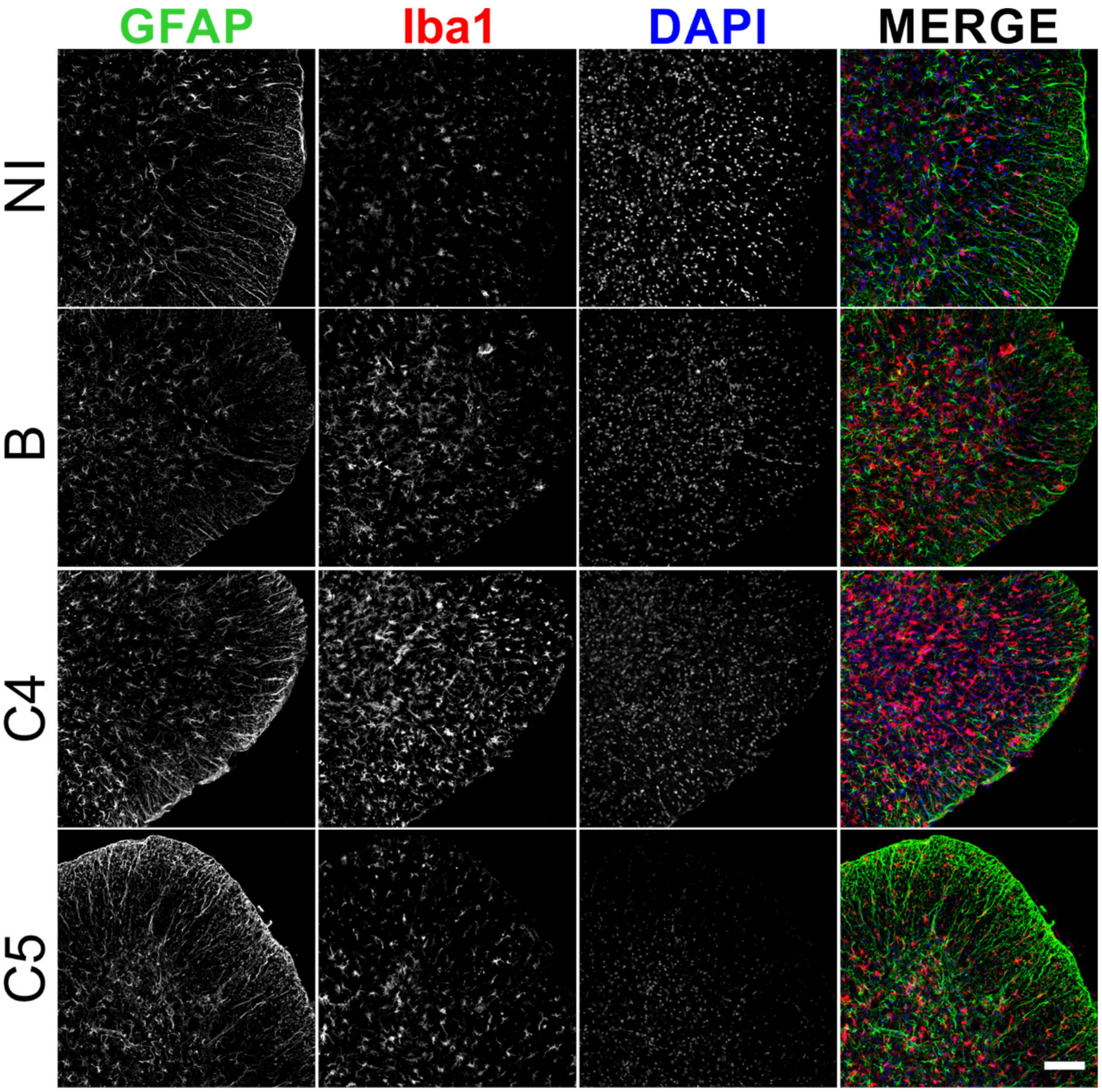

Figure S3: C4 and C5 treatment had no effect on the neuroinflammatory response in SOD1^{G93A} mice spinal cords. Immunofluorescence staining of activated astrocytes (GFAP) and activated microglia (Iba1) in lumbar spinal cord sections of SOD1^{G93A} mice. Scale bar= 100μm.

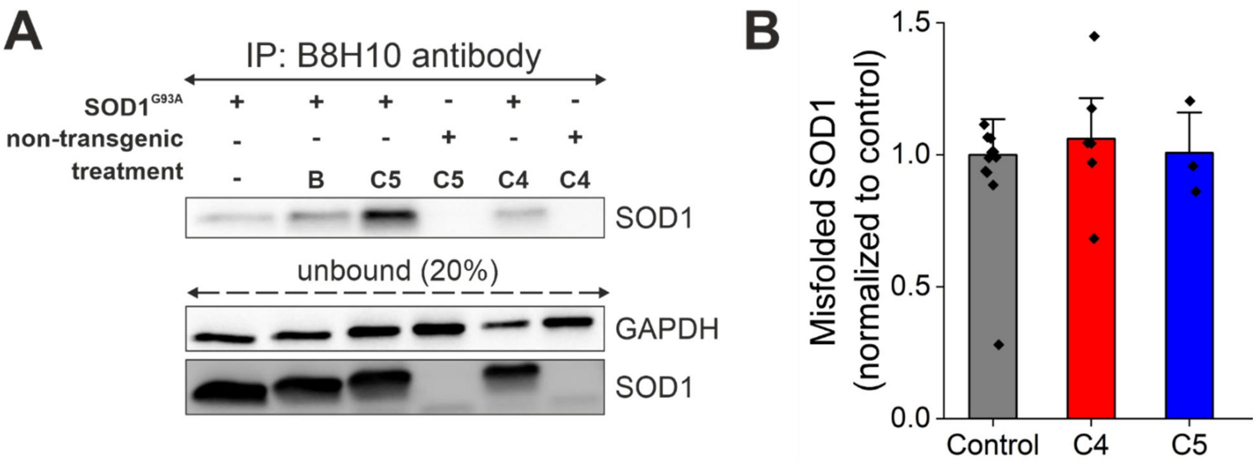

Figure S4: C4 and C5 chemical chaperones did not reduce SOD1 misfolding *in vivo*. (A) Misfolded-SOD1 levels were detected by immunoblotting of immunoprecipitates produced with B8H10 antibody, from SOD1^{G93A} mice brains. IP- immunoprecipitation; B- acetate buffer. **(B)** Quantification of misfolded-SOD1 levels in untreated (grey) and C4 (red) and C5 (blue) treated SOD1^{G93A} mice.

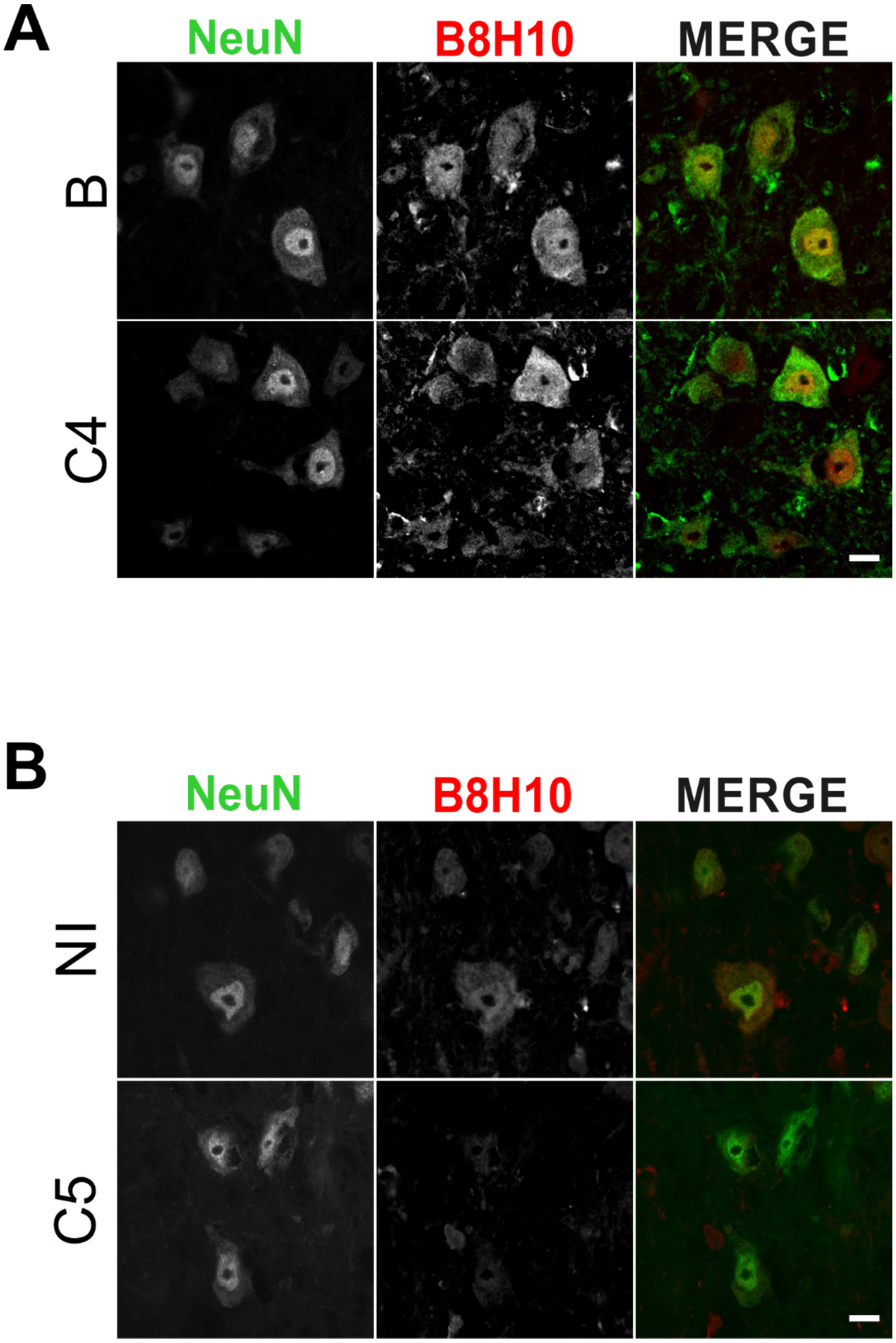

Figure S5: C4 and C5 treatment had no effect on the accumulation of misfolded SOD1 in the spinal cord of mutant SOD1^{G93A} mice. (A, B) Immunofluorescence staining of motor neurons (NeuN) and misfolded SOD1 (B8H10) in lumbar spinal sections of C4 **(A)** and C5-treated **(B)** mice and untreated SOD1^{G93A} mice. Scale bar= 10µm.

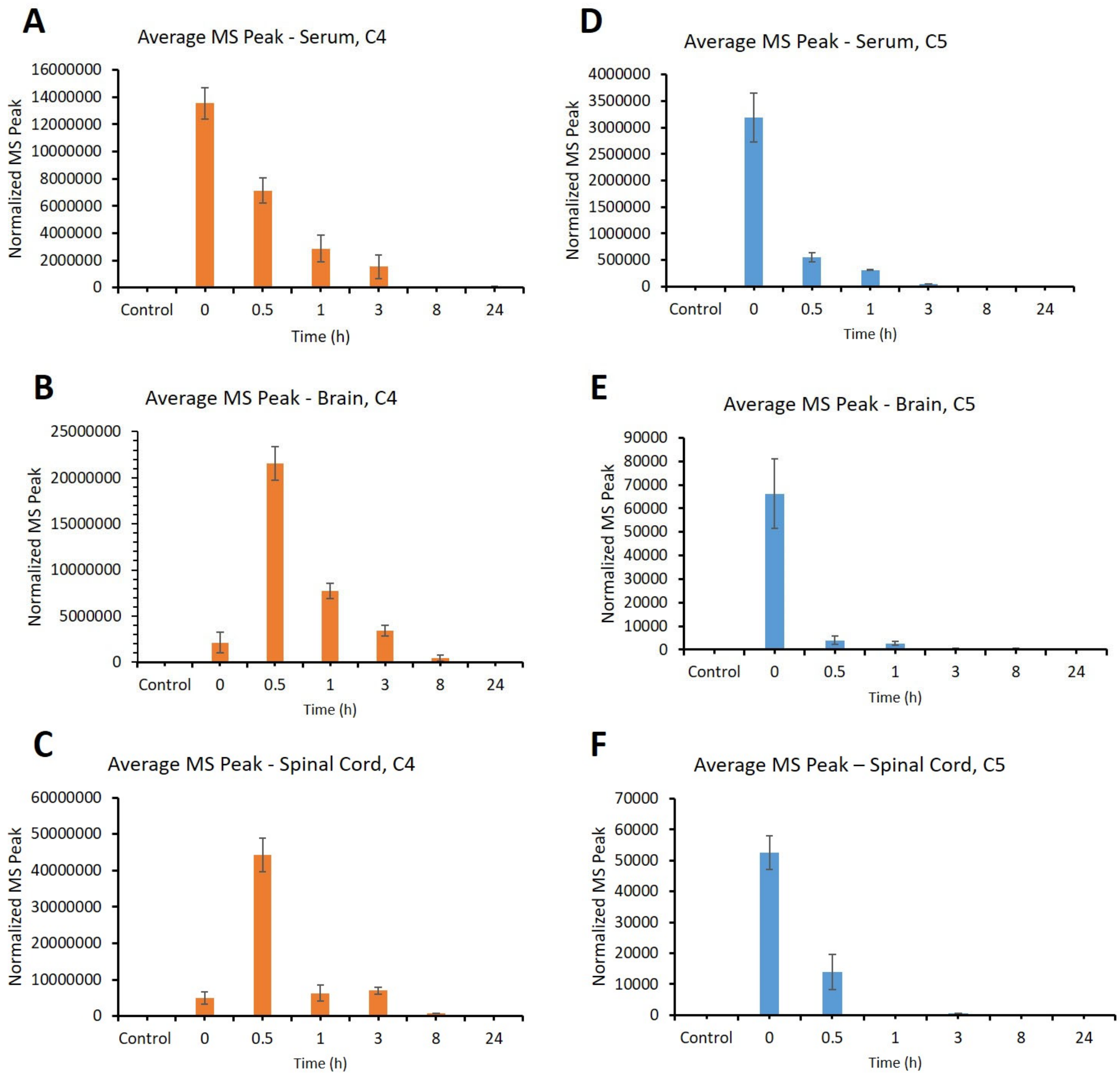

Figure S6. LC-MS PK results of the determination of the levels of C4 and C5. PK results show the levels of C4 (**A-C**) and C5 (**D-F**) in serum (**A, D**), brain (**B, E**) and spinal cord (**C, F**).
